# Getting to Grips with the Oxysterol-Binding Protein Family – a Forty Year Perspective

**DOI:** 10.1177/25152564241273598

**Published:** 2024-08-28

**Authors:** Vesa M. Olkkonen, Elina Ikonen

**Affiliations:** 1 Minerva Foundation Institute for Medical Research, Helsinki, Finland; 2 Faculty of Medicine, Dept of Anatomy and Stem Cells and Metabolism Research Program, University of Helsinki, Helsinki, Finland

**Keywords:** lipid transfer protein, lipid transport, membrane contact site, OSBP, protein family

## Abstract

This review discusses how research around the oxysterol-binding protein family has evolved. We briefly summarize how this protein family, designated OSBP-related (ORP) or OSBP-like (OSBPL) proteins, was discovered, how protein domains highly conserved among family members between taxa paved the way for understanding their mechanisms of action, and how insights into protein structural and functional features help to understand their versatility as lipid transporters. We also discuss questions and future avenues of research opened by these findings. The investigations on oxysterol-binding protein family serve as a real-life example of the notion that science often advances as a collective effort of multiple lines of enquiry, including serendipitous routes. While original articles invariably explain the motivation of the research undertaken in rational terms, the actual paths to findings may be less intentional. Fortunately, this does not reduce the impact of the discoveries made. Besides hopefully providing a useful account of ORP family proteins, we aim to convey this message.

## Introduction

The OSBP-related (ORP/OSBPL) protein family is present throughout the eukaryotic kingdom. These proteins, initially assumed to act as oxysterol sensors with crucial roles in the maintenance of cellular sterol homeostasis, turned out to act as lipid transporters over a range of membrane contact sites (MCS) between distinct subcellular organelles. Of note, their lipid cargoes are not limited to sterols, but they also transfer phosphatidylinositol phosphates (PIPs), phosphatidylserine (PS), and perhaps other, as yet unidentified lipid ligands. A number of ORPs are suggested to mediate the transport of lipids against their intracellular concentration gradients by employing countertransport of another lipid down its concentration gradient. However, certain family members may execute functions beyond lipid transfer: ORPs are also suggested to play roles in signaling cascades and gene regulation. The functions of ORPs have been extensively covered in recent review articles (Antonny et al., 2018; Arora et al., 2022; Olkkonen and Ikonen, 2022; Pietrangelo and Ridgway, 2018; Subra et al., 2023). Therefore, in this perspectives-type review we do not aim to exhaustively summarize the work carried out on ORPs, but rather attempt to cast light on some of the key discoveries that in our opinion have led to conceptual break-throughs. Moreover, we sketch tentative future directions in the study of the ORP/OSBPL family. We apologize to authors whose work is not cited due to space limitations and the selective nature of this article.

### Oxysterol-Binding Protein is not a major Transcriptional Regulator of Sterol Homeostasis

Early observations had shown that 27-carbon oxygenated derivatives of cholesterol, or oxysterols, act as potent suppressors of cholesterol biosynthesis (Kandutsch et al., 1978). This prompted a search for proteins with the capacity to mediate the effects of oxysterols on cholesterol/lipid metabolism. Protein fractions with oxysterol-binding activity were isolated from various biological sources (Defay et al., 1982; Kandutsch et al., 1977; Kandutsch and Shown, 1981). F.R. Taylor, E.P. Shown, A.A. Kandutsch and co-workers eventually identified an oxysterol receptor whose affinity for different sterols correlated with their potency to suppress 3-hydroxy-3-methylglutaryl coenzyme A reductase (HMGCR), a rate-limiting enzyme of cholesterol biosynthesis, and named it oxysterol-binding protein (OSBP)(Taylor and Kandutsch, 1985; Taylor et al., 1984).

The group of M.S. Brown and J.L. Goldstein (Univ of Texas Southwestern, Dallas, USA) got interested in OSBP in search of a controller of sterol homeostatic genes. Their laboratory managed to purify this protein (Dawson et al., 1989b) followed by cloning of the OSBP cDNA (Dawson et al., 1989a; Levanon et al., 1990). However, Brown and Goldstein soon realized that this protein is not a regulator of sterol homeostatic gene transcription. N.D. Ridgway working as a post-doctoral researcher in their group found that OSBP does not localize within the nucleus but rather in the cytoplasm, and treatment of cells with the commonly used oxysterol 25-hydroxycholesterol (25-HC) induced its translocation to the Golgi complex (Ridgway et al., 1992).

The discovery of sterol regulatory element binding proteins (SREBPs)(Hua et al., 1993; Wang et al., 1993; Yokoyama et al., 1993) and liver X receptors (LXRs)(Janowski et al., 1996; Lehmann et al., 1997) as major transcriptional regulators of sterol metabolism soon directed the mainstream of research away from OSBP. However, studies on OSBP were systematically continued by the group of N.D. Ridgway (Dalhousie University, Halifax, Canada) (Pietrangelo and Ridgway, 2018; Ridgway, 2010), and later on, some OSBP family members were actually reported to execute nuclear functions (see Concluding remarks).

### Discovery of OSBP Homologues – the Idea of Functional Redundancy

The first OSBP-related proteins were identified in the early 1990s in the yeast *Saccharomyces cerevisiae*. Schmalix and Bandlow (Schmalix and Bandlow, 1993) initially cloned and sequenced yeast mitochondrial carnitine acetyltransferase YAT1, finding in the same genomic fragment another gene they called SWI4/6-related gene, SWH1, due to the presence of ankyrin repeats in the encoded protein. The next year they characterized SWH1 further, reporting it as a non-essential gene encoding an OSBP-related protein (Schmalix and Bandlow, 1994), while (Jiang et al., 1994) identified three yeast genes with homology to OSBP, which they called KES1, HES1 and OSH1. Mutations in these genes resulted in pleiotropic sterol-related phenotypes. A further interesting study by the group of V.A. Bankaitis (University of Alabama, Birmingham, USA) reported a function of KES1 in the biogenesis of Golgi-derived transport vesicles (Fang et al., 1996).

Soon after, the groups of E. Ikonen and V. Olkkonen (National Public Health Institute, Helsinki, Finland), in search for the Niemann-Pick C1 (NPC1) gene that had been mapped to the proximal region of chromosome 18q, stumbled onto additional human homologues of OSBP and named this family OSBP related proteins, ORPs (Laitinen et al., 1999). Fortuitously, the ORP1/OSBPL1A gene in 18q is located only 575 kb upstream of the NPC1 gene in the same strand. The search of human expressed sequence tags then revealed a minimum of six novel ORPs (Laitinen et al., 1999), followed by a systematic characterization of the entire human 12-member ORP family in human (Jaworski et al., 2001; Lehto et al., 2001) and mouse (Anniss et al., 2002), in which the genes are named OSBP and OSBPL1-11. Related genes (4 in each organism) were also discovered in *C. elegans* (Kobuna et al., 2010; Sugawara et al., 2001) and *D. melanogaster* (Alphey et al., 1998; Ma et al., 2010).

Importantly, in 2001 the group of J. Rine (University of California, Berkeley) reported an exhaustive study on the entire 7-member yeast OSBP-related gene family and established the nomenclature of yeast OSBP homologs (Osh) as OSH1-7 (Beh et al., 2001). The previously studied KES1 is in this current nomenclature OSH4, HES1 is OSH5 and SWH1 is OSH1. Beh et al. constructed all 127 combinations and permutations of OSH deletion alleles and showed that individual OSH genes were not essential for yeast viability, but the elimination of the entire gene family was lethal. The authors also observed that depletion of all Osh proteins disrupted sterol homeostasis, providing evidence that, even though OSBP and Osh proteins do not act as transcriptional regulators of sterol homeostatic gene expression, their functions do significantly reflect on sterol homeostasis.

Although none of the single OSH deletion mutants was defective for growth, gene expression profiles revealed that each mutant had a characteristic molecular phenotype. The authors thus concluded that each Osh protein performs distinct nonessential functions and contributes to a common essential function, and that there may exist a degree of functional redundancy between the family members. This concept has had a significant impact on the study of OSBP homologues also in other taxa, including human and mouse. A tentative functional redundancy has been thought to provide an explanation to the fact that the reported mammalian ORP8, −2 and −4 knock-out (KO) mouse phenotypes are mild (Beaslas et al., 2013; Koponen et al., 2020; Udagawa et al., 2014). There is a long-standing rumor of unpublished results by the Brown and Goldstein group showing that OSBP KO in mice is embryonic lethal. So far, there seems to be no evidence that other mammalian ORPs would be essential − further study is required to clarify this.

### Dual Membrane Targeting Sparks the Idea of ORP Function at Membrane Contact Sites

When T. Levine and S. Munro (Levine and Munro, 2001) investigated the subcellular localization of yeast Osh1p, they discovered that it targets both the late Golgi apparatus and the nucleus-vacuole junction (NVJ), a membrane contact site (MCS) characteristic of yeast (Kvam and Goldfarb, 2006). This was the first report of a MCS localization of an ORP. Deletion mapping revealed that the pleckstrin homology (PH) domain of Osh1p specified targeting to the late Golgi, and an ankyrin repeat domain in the amino-terminal region of the protein targeting to the NVJ.

The same investigators had in 1998 found that the pleckstrin homology (PH) domains of both yeast Osh1p and mammalian OSBP target the Golgi complex mediated by interactions with membrane phosphoinositides (Levine and Munro, 1998) and with the small GTPase Arf1 (Levine and Munro, 2002). These observations formed the conceptual background for the groundbreaking report of (Loewen et al., 2003), where T. Levine's group (University College London, UK) identified a short, conserved motif designated two phenylalanines in an acidic tract (FFAT). This motif with a consensus sequence EFFDAxE was shared by seven mammalian OSBP/ORP family members (OSBP, ORP1L, 2, 3, 4, 6, 7, and 9) as well as yeast Osh1-3p, and was found to interact with the major sperm protein (MSP) domain of the integral endoplasmic reticulum (ER) proteins VAPA and VAPB, or their yeast orthologue Scs2p. Of note, the four mammalian ORPs lacking this domain exhibit alternative ways of membrane targeting: ORP5 and −8 carry an ER-targeting transmembrane segment in their carboxy-terminal region (Du et al., 2011; Yan et al., 2008), and ORP10 and −11 can target membranes via dimerization with ORP9, in addition to PH domain interactions with membrane phosphoinositides (He et al., 2023; Kawasaki et al., 2022; Naito et al., 2023; Zhou et al., 2010).

Discovery of the FFAT motif provided a mechanistic explanation to the observed ER targeting of several ORPs. Together with the observations that the amino-terminal PH domains of ORPs target phosphoinositide-rich membranes, such as Golgi, plasma membrane or endosomes, this implied that ORPs might employ a dual targeting mechanism (via the FFAT motif and the PH domain) to shuttle between two organelle membranes, or even bind two membranes simultaneously. Obviously, localization of an ORP or other lipid transfer protein at a MCS would allow it to carry out very precise and rapid intercompartmental lipid transfer, avoiding the cycles of association and dissociation from the membranes required by a shuttle-type action.

V. Olkkonen and T. Levine discussed these ideas at the 44th International Conference on the Bioscience of Lipids, Oxford, UK, in September 2003, and wrote an article putting forward the hypothesis of ORP function at MCSs (Olkkonen and Levine, 2004), which subsequent research has validated. Moreover, additional mammalian VAP-related proteins (Cabukusta et al., 2020; Thaler et al., 2011) and versions of the FFAT motif [FFAT-like, two phenylalanines in a neutral tract (FFNT) and phospho-FFAT motifs] have been discovered (James and Kehlenbach, 2021; Mikitova and Levine, 2012), and over 250 proteins have been found to bind to VAPs (Cabukusta et al., 2020; Huttlin et al., 2021; Huttlin et al., 2017; Huttlin et al., 2015; James et al., 2019). The VAP family proteins have thereby emerged as major organizers of ER MCSs recruiting FFAT- or FFAT-like motif carrying protein components, including ORPs, at these sites.

### ORPs are not Exclusively Sterol Binders but Also Bind Glycerophospholipids – ORPs as Countercurrent Lipid Transporters

Structural studies of yeast Osh4p by the group of J. Hurley (National Institutes of Health, Bethesda, MD) revealed that the OSBP-related ligand-binding domain (ORD) of ORPs can accommodate various sterols, including oxysterols, ergosterol and cholesterol (Im et al., 2005). At the same time, the group of W. Prinz (National Institutes of Health, Bethesda, MD) reported that the yeast Osh proteins are required for sterol transport from the plasma membrane to the ER (Raychaudhuri et al., 2006). Importantly, they also noted that Osh4p transfers sterols more rapidly between membranes containing phosphoinositides (PIPs), suggesting that PIPs regulate sterol transport by ORPs. The groups of B. Antonny and G. Drin (Université de Nice Sophia-Antipolis and CNR, Valbonne, France) then made the crucial observation that the glycerophospholipid phosphatidylinositol 4-phosphate (PI4P) inhibited the extraction of dehydroergosterol (DHE) from membranes by Osh4p (de Saint-Jean et al., 2011). They not only solved the structure of Osh4p with PI4P bound to its ORD ligand-binding pocket, but suggested that Osh4p quickly exchanges DHE for PI4P, potentially transporting these two lipids between membranes in opposite directions.

**Figure 1.**
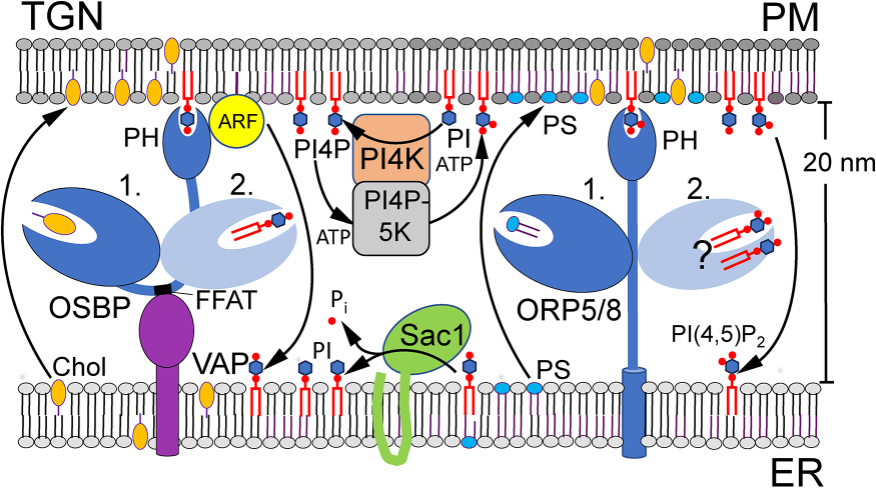
The principle of countercurrent lipid transfer by OSBP/ORPs at MCSs. On the left the OSBP-mediated transfer of cholesterol (Chol) and phosphatidylinositol 4-phosphate (PI4P) over ER-*trans*-Golgi network (TGN) contact sites is depicted. On the right ORP5/8-mediated phosphatidylserine (PS) and PI4P or PI(4,5)P_2_ exchange over ER-plasma membrane (PM) contacts is displayed. The PI4P arriving at the ER is hydrolyzed by the phosphatase Sac1. Other abbreviations: ARF, ADP-ribosylation factor (a small GTPase); FFAT, two phenylalanines in an acidic tract motif; PH, pleckstrin homology domain; PI, phosphatidylinositol; P_i_, inorganic phosphate; PI4 K, PI-4-kinase; PI4P-5 K, PI4P-5-kinase; VAP, VAMP-associated protein. Modified from (Arora et al., 2022).

This finding turned out to be an icebreaker in the elucidation of the mechanism of ORPs in intracellular lipid transfer. Later on, the Antonny and Drin groups demonstrated that both mammalian OSBP (Mesmin et al., 2013) as well as yeast Osh4p (Moser von Filseck et al., 2015b) and Osh6p (Moser von Filseck et al., 2015a) are able to carry out so-called lipid countercurrent transport (Figure 1). Here, ORPs employ the energy deposited in a gradient of PI4P between late and early compartments of the secretory pathway to drive the forward transport of cholesterol (OSBP), ergosterol (Osh4p) or phosphatidylserine (PS, Osh6p) from the ER, against their concentration gradients, towards the *trans*-Golgi or the plasma membrane (PM).

In the countercurrent transport model, an important factor maintaining the intercompartmental PI4P gradient is the phosphatase Sac1 that hydrolyses PI4P arriving in the ER via the lipid exchange activity of ORPs (Figure 1). Of note, the capacity of Osh6p and Osh7p to bind PS and transport it from the ER to the PM had been demonstrated by the group of A.-C. Gavin (European Molecular Biology Laboratory, EMBL, Heidelberg, Germany), who also provided the first evidence that the mammalian ORP5 and −10 act as PS transporters (Maeda et al., 2013). The PS/PI4P countercurrent transfer activity of mammalian ORP5 and the closely related ORP8 at ER-PM contacts (Chung et al., 2015) (Figure 1) was soon demonstrated by the group of P. De Camilli (Yale School of Medicine, New Haven, CT). Later on, it was shown that ORP5 and −8 also localize and function at ER-mitochondrial contacts (Galmes et al., 2016; Monteiro-Cardoso et al., 2022).

These hallmark studies pushed forward the concept of a bidirectional countercurrent lipid transfer mechanism executed by lipid transfer proteins (LTPs) at MCSs. Subsequent observations on multiple ORPs have lent further support to this concept (Table 1). Moreover, there is substantial evidence that also other phosphoinositides than PI4P can act as ORP cargoes (Ghai et al., 2017; Takahashi et al., 2021; Wang et al., 2019) and that also other LTPs can employ the countercurrent transport principle, as exemplified by Nir2/PITPNM1, which carries out PI/phosphatidic acid (PA) exchange at ER-PM contacts (Kim et al., 2015).

**Table 1.** Countercurrent Transport of Lipids by ORPs.

Protein	Lipid ligands	Organelles/MCS	Reference(s)
Osh4p	Ergosterol, PI4P	ER-Golgi	(de Saint-Jean et al., 2011; Moser von Filseck et al., 2015b)
Osh6p	PS, PI4P	ER-PM	(Moser von Filseck et al., 2015a)
OSBP	Cholesterol, PI4P	ER-TGN	(Mesmin et al., 2013)
		ER-lysosome	(Lim et al., 2019; Radulovic et al., 2022)
		ER-insulin secretory granule	(Panagiotou et al., 2024)
ORP2	Cholesterol, PI(4,5)P_2_	LE-RE-PM	(Takahashi et al., 2021; Wang et al., 2019)
ORP5/8	PS, PI4P, PI(4,5)P_2_	ER-PM	(Chung et al., 2015; Ghai et al., 2017; Sohn et al., 2018)
ORP10	PS, PI4P	ER-endosome	(Kawasaki et al., 2022)
		ER-damaged lysosome	(Tan and Finkel, 2022)
	PS, PI4P	ER-TGN	(He et al., 2023; Venditti et al., 2019)
ORP11	PS, PI4P	ER-damaged lysosome	(Tan and Finkel, 2022)

Abbreviations: MCS, membrane contact site; PI4P, phosphatidylinositol 4-phosphate; ER, endoplasmic reticulum; PS, phosphatidylserine; PM, plasma membrane; TGN, *trans*-Golgi network; LE, late endosome; RE, recycling endosome; PI(4,5)P_2_, phosphatidylinositol 4,5-bisphosphate.

### High-Resolution Structures of ORPs Yield Insights into Their Functional Mechanisms

**Figure 2.**
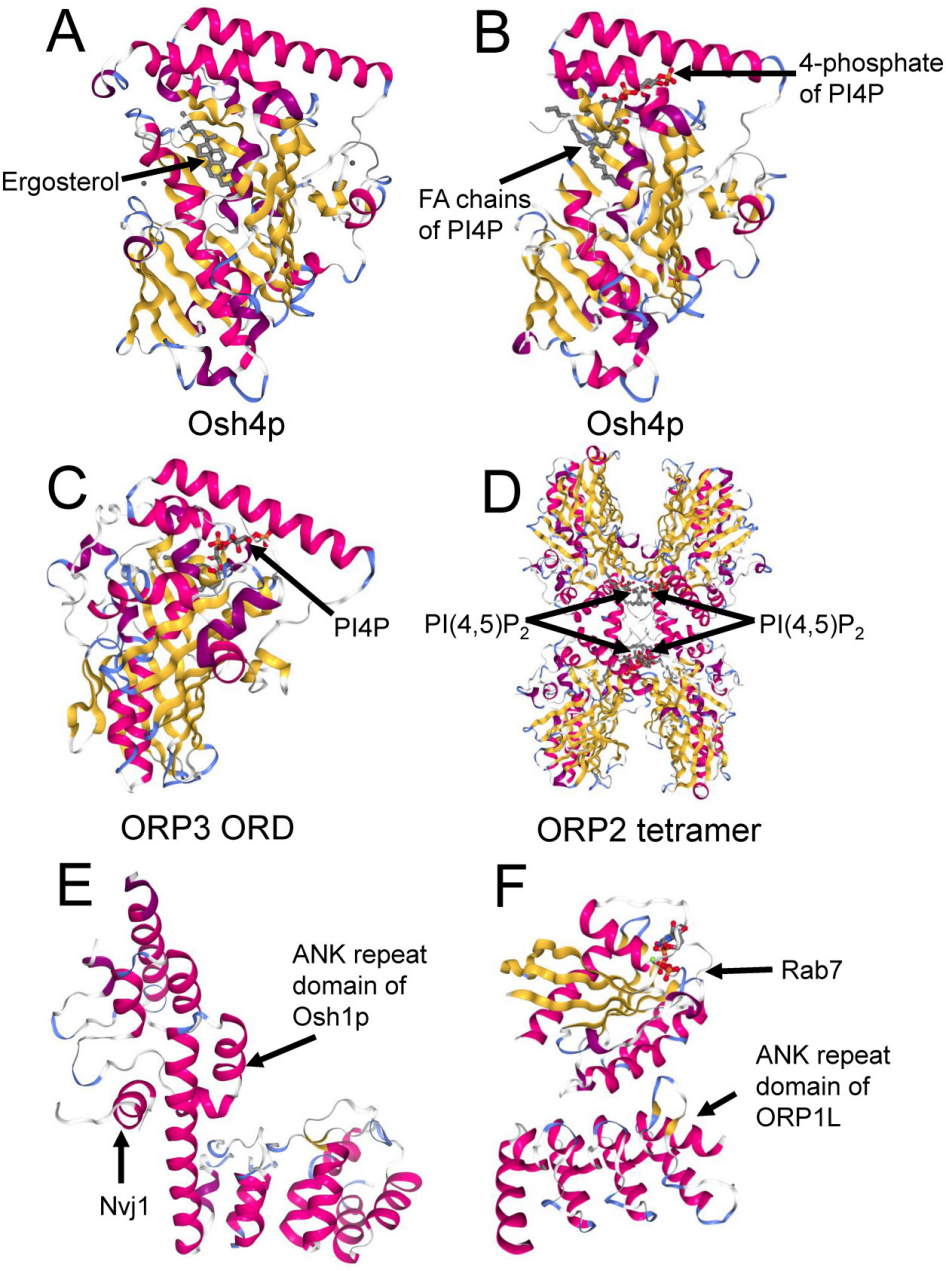
Structures of selected ORPs with bound lipid ligands and complexes of ORPs with interacting proteins. A. Yeast Osh4p with bound ergosterol (PDB: 1ZHZ). B. Osh4p with bound PI4P (3SPW). C. Human ORP3 ORD with bound PI4P (7DEI). D. Human ORP2 tetramer with bound PI(4,5)P_2_ (5ZM8). E. Yeast Osh1p ankyrin repeat domain (ANK) complexed with Nvj1p (5H2C). F. Human ORP1L ANK domain complexed with the small GTPase Rab7 (6IYB).

While the ORD accommodates both sterols and phospholipids, the mechanisms by which ligands interact with the binding pocket differ. The first structural study of yeast Osh4p (Im et al., 2005) found that a single sterol molecule binds within the hydrophobic tunnel of the ORD, with the 3-hydroxyl moiety directed towards the bottom of the tunnel (Figure 2A). The entrance is blocked by a flexible amino-terminal lid and surrounded by basic residues critical for Osh4p function. Sterol binding closes the lid and stabilizes a conformation favoring transport of the bound sterol across aqueous barriers. The structure of Osh4p in the absence of ligand exposes potential phospholipid-binding sites that are positioned for membrane docking and sterol exchange.

Regarding phosphoinositide binding, (de Saint-Jean et al., 2011) discovered that charged amino acid residues defining a shallow pocket under the lid of Osh4p recognize the PI4P head group, and the PI4P fatty acyl chains loosely interact with the sterol binding cavity in a rather non-specific manner (Figure 2B). The histidines in the conserved OSBP fingerprint motif EQVSHHPP were found to interact with the polar head group of PI4P, suggesting that all ORPs might be capable of binding PI4P.

Since the initial Osh4p structure, high-resolution structures of the ORDs of several mammalian and yeast ORPs with bound sterols, PI4P, PI(4,5)P_2_ or PS have been solved (Dong et al., 2019; Dong et al., 2020; Eisenreichova et al., 2023; Kobayashi et al., 2022; Maeda et al., 2013; Manik et al., 2017; Moser von Filseck et al., 2015a; Tong et al., 2021; Tong et al., 2013; Wang et al., 2019). Structural studies suggest that the PI4P binding of ORPs is strictly conserved but sterol binding is not. For instance, Tong and coworkers (Tong et al., 2013) reported that Osh3p lacks sterol binding due to the narrow hydrophobic tunnel, and the same was observed for human ORP3 (Tong et al., 2021) (Figure 2C).

Interestingly, studies by the group of H. Yang (University of New South Wales, Sydney, Australia) on human ORP1 (Dong et al., 2019) and ORP2 (Wang et al., 2019) demonstrated that the ORD lipid-binding tunnel can also accommodate phosphoinositides other than PI4P, at least PI(4,5)P_2_, consistent with the observation that ORP2 is capable of the countercurrent transfer of cholesterol and PI(4,5)P_2_ (Takahashi et al., 2021; Wang et al., 2019). Of note, the PI(4,5)P_2_-bound ORP2 formed a tetramer (Wang et al., 2019) (Figure 2D), suggesting not only that the liganding of ORPs may regulate their oligomeric state but also that oligomerization may affect the choice of ligand. Whether ORP1L is capable of cholesterol-phosphoinositide countercurrent transport remains open. While Dong et al. (Dong et al., 2019) could not detect transport of any PIP by ORP1L, the study of (Levin-Konigsberg et al., 2019) suggested that it transports PI4P from phagolysosomes to the ER. However, in this study cholesterol transport or putative PI4P-cholesterol countertransport were not addressed.

Structural information on ORPs in complex with other proteins is still scarce but two structures of ORP ankyrin repeat (ANK) domain complexes have been reported. Manik and coworkers (Manik et al., 2017) determined the structure of the Osh1p ANK domain in complex with the nucleus-vacuole junction protein Nvj1 (Figure 2E). The Osh1p ANK forms a unique bi-lobed structure that recognizes a cytosolic helical segment of Nvj1, mediating the localization of Osh1p at the NVJ. Tong and coworkers (Tong et al., 2019) determined the crystal structure of human ORP1 ANK domain in complex with the GTP-bound form of the late endosomal GTPase Rab7 (Figure 2F). ORP1 ANK bound to the helix α3 of Rab7 located away from the nucleotide-dependent switch regions, making the interaction independent of the nucleotide-binding state of Rab7.

### How are the Functions of ORPs Regulated in the Cellular Context?

In the fluctuating conditions of cell physiology, the needs for inter-organelle lipid transport vary considerably. This necessitates intricate regulation of LTPs, including ORPs. Below, we discuss selected aspects of how the direction of lipid transfer may be dictated and how ORPs themselves maybe be regulated, e.g., by phosphorylation or oligomerization, to modulate function.

#### Role of Phosphoinositide Gradients in Driving Lipid Transfer and its Directionality

Mesmin and coworkers (Mesmin et al., 2013) initially revealed how PI4P in the *trans*-Golgi membranes, recognized by the PH domain of OSBP, contributes to the localization of OSBP at ER-*trans*-Golgi contact sites, and how its reduction upon lipid transfer mediated by OSBP acts as a feedback regulatory signal, detaching OSBP from the MCSs. In a similar manner, the PI4P generated by the PI-4-kinase PI4K2A on damaged lysosomes is bound by the OSBP PH domain, contributing to the generation of ER-lysosome contacts over which cholesterol is transported to patch the damaged lysosomal membrane (Radulovic et al., 2022; Tan and Finkel, 2022).

Phosphoinositide gradients regulate the function of other ORPs as well: A good example are ORP5 and ORP8, whose PH domains bind plasma membrane PI(4,5)P_2_ and PI4P for recruitment to sites where they mediate the countercurrent transfer of PS against phosphoinositides (Ghai et al., 2017; Sohn et al., 2018). In this way ORP5/8 control the relative flux of PI4P toward the ER for Sac1-mediated dephosphorylation vs. the PM for conversion to PI(4,5)P_2_. Of note, phosphoinositides may also exert allosteric regulation of lipid transporters, as demonstrated for ORP1L: PI(4,5)P_2_ and PI(3,4)P_2_ bound to the ORP1L ORD allosterically enhance ORP1L-mediated cholesterol transport (Dong et al., 2019).

#### Role of Organelle Cholesterol Concentration and Modifying Protein Interactors

An intriguing aspect of ORP regulation is that at least certain family members may transfer a lipid substrate in opposite directions under different physiologic conditions. A good example is ORP1L at ER-late endosome (LE)/lysosome (Lys) MCSs. The protein interacts with VAPs in the ER and with the small GTPase Rab7 in the LE/Lys membranes. Zhao and Ridgway (Zhao and Ridgway, 2017) provided evidence that knock-out of ORP1L in HeLa or HEK293 cells caused the accumulation of cholesterol-enriched LEs/Lys and reduced cholesterol esterification by 60–80%, suggesting a defect in cholesterol transport from Lys to the ER. In agreement, Höglinger and coworkers (Hoglinger et al., 2019) demonstrated that ORP1L overexpression dramatically increased ER-lysosome contacts and reduced lysosomal cholesterol accumulation in NPC1-null cells. However, the cholesterol accumulation was not only rescued by wild-type ORP1L but also by ORP1L ΔORD lacking the sterol-binding domain, suggesting that the rescue may rather reflect the ability of ORP1L to expand the LE/Lys-ER MCSs than to act as a cholesterol transporter.

On the other hand, Kobuna and coworkers (Kobuna et al., 2010) first reported that knocking down ORP1L in HeLa cells induced the formation of enlarged LEs/multivesicular bodies (MVBs) lacking the normal content of intraluminal vesicles (ILVs), similar to those formed upon cholesterol restriction of *C. elegans*. This indicated that ORP1L may mediate cholesterol transport from the ER to LEs, a conclusion supported by Eden and coworkers (Eden et al., 2016), who demonstrated a function of ORP1L in the transfer of ER-derived cholesterol to the LE at Annexin A1 (AnxA1)-regulated ER-LE contacts. This cholesterol traffic is apparently required for the formation of ILVs within the LEs/MVBs.

How the direction of ORP-mediated lipid transfer is determined requires further studies. In the case of ORP1L, the cholesterol concentration in the LE/MVB/Lys membrane may play a critical role, since sterol binding of ORP1L controls the conformation of the protein and its affinity for VAPs, and possibly other protein interactors (Rocha et al., 2009; Vihervaara et al., 2011). The role of protein-protein interactions is also supported by Cianciola and coworkers (Cianciola et al., 2013), who showed that the adenoviral protein RIDα rescues lysosomal cholesterol storage in *NPC1* mutant fibroblasts: the interaction of ORP1L with RIDα reconstitutes the deficient endosome-to-ER transport of cholesterol.

#### Regulation of ORPs by Phosphorylation and Ions

The extensive work by N. Ridgway and coworkers has demonstrated the crucial role of phosphorylation in regulating the function of OSBP. This modification, at least in part catalyzed by protein kinase D, occurs at the Golgi complex and regulates the interactions of OSBP with sterols and VAPA (Goto et al., 2012; Nhek et al., 2010; Ridgway et al., 1998; Storey et al., 1998). Pietrangelo and Ridgway (Pietrangelo and Ridgway, 2019) also observed that a unique proline/serine-rich phosphorylation motif in the ORD of the closest homologue of OSBP, ORP4L, induces a conformational change that enhances its interaction with vimentin and cholesterol extraction from membranes.

Yet another ORP family member regulated by phosphorylation is ORP3. We showed that hyperphosphorylated ORP3 selectively interacts with VAPA, and ORP3-VAPA complexes are targeted to PM sites via the ORP3 PH domain. Co-expression of ORP3 and VAPA induced R-Ras activation, dependent on the interactions of ORP3 with VAPA and the PM, plausibly at ER-PM contact sites (Weber-Boyvat et al., 2015). Consistent with our data, the group of T. Balla (National Institutes of Health, Bethesda, MD) showed that the PM targeting of ORP3 is triggered by protein kinase C (PKC) activation, especially in combination with Ca^2+^ increase, and is determined by both PI(4,5)P_2_ and PI4P at the PM. Full activation of ORP3 resulted in decreased PM PI4P levels and inhibited store-operated Ca^2+^ entry (Gulyas et al., 2020).

The pH-dependent protonation of phosphoinositides may also regulate ORP/Osh function. Shin and coworkers (Shin et al., 2020) demonstrated that binding of PH domains to PI4P in the yeast TGN depends on the pH. The data suggested that pH biosensing by TGN PI4P in response to nutrient availability governs protein sorting at the TGN, likely by regulating sterol transfer to the TGN by Osh1p. A further regulatory mechanism was revealed by Malek et al. (Malek et al., 2021) showing that calcium efflux from ER stores induced by inositol-triphosphate (IP_3_) resulted in the dissociation of OSBP from the Golgi complex and from VAP-containing MCSs. This triggered depletion of cholesterol and associated globoside 3 (Gb3) from the cell surface, resulting in a blockade of Shiga toxin endocytosis.

#### Regulation of ORPs by Oligomerization

Several ORPs are known to form homo- or heterodimers or oligomers, but the precise functional or regulatory roles of oligomerization are not well understood. OSBP can function as a homodimer (de la Mora et al., 2021; Ridgway et al., 1992) or heterodimer with the closely related ORP4L (Wyles et al., 2007; Zhong et al., 2022). The ligand-free ORP1 ORD formed mono-, di- or trimers, with cholesterol binding shifting the distribution towards monomers (Dong et al., 2019). Also cholesterol-bound ORP2 appeared as a monomer while PI(4,5)P_2_-bound ORP2 was tetrameric (Wang et al., 2019).

Heterodimerization of closely related ORPs, summarized in Table 2, has emerged as a regulatory mechanism. Whether higher order oligomeric assemblies of ORPs than dimers function at MCSs remains to be investigated. Interestingly, recent work suggests that ORPs can function as part of supramolecular complexes mediating intracellular lipid fluxes. Anwar and coworkers (Anwar et al., 2022) identified TMED2 and TMED10 as essential components of a supercomplex that operates the exchange of both cholesterol and ceramides at ER-Golgi MCSs. These complexes also contained OSBP, VAPA and the ceramide transporter CERT, playing a role in the formation of plasma membrane lipid nanodomains. Such higher order complexes containing ORPs are likely to operate at MCSs and should be delicately regulated through the abundance, assembly and post-translational modifications of their components.

**Table 2.** Heterodimers Formed by Closely Related ORPs.

Proteins	Localization/MCS	Function	References(s)
OSBP-ORP4L	?	Vimentin organization	(Wyles et al., 2007)
	TGN-PM	Transport of PI4P to the PM	(Zhong et al., 2022)
ORP3-ORP6	ER-PM	PI4P distribution	(Mochizuki et al., 2018)
ORP5-ORP8	ER-PM	PS-PI4P/PI(4,5)P_2_ exchange	(Chung et al., 2015; Ghai et al., 2017; Sohn et al., 2018)
	ER-mitochondria	Transport of PS to mitochondria	(Galmes et al., 2016; Monteiro-Cardoso et al., 2022)
	MAM-LD	Lipid droplet biogenesis	(Guyard et al., 2022)
ORP9-ORP10	Golgi	?	(Nissilä et al., 2012)
	ER-endosome	PS-PI4P counter-transport	(Kawasaki et al., 2022)
	ER-TGN	PI4P homeostasis at ER-TGN MCS and vesicle trafficking	(He et al., 2023)
ORP9-ORP11	Golgi	?	(Zhou et al., 2010)
ORP9-ORP10/ORP11	ER-TGN	Regulation of Golgi PI4P and (indirectly) cholesterol	(Naito et al., 2023)

Abbreviations: MCS, membrane contact site; TGN, *trans*-Golgi network; PM, plasma membrane; PI4P, phosphatidylinositol 4-phosphate; ER, endoplasmic reticulum; PS, phosphatidylserine; PI(4,5)P_2_, phosphatidylinositol 4,5-bisphosphate; MAM, mitochondria-associated membranes; LD, lipid droplet; TGN, *trans*-Golgi network.

### ORPs as Emerging Drug Targets

The era of pharmacological inhibition of OSBP/ORPs was initiated by Burgett and coworkers (Burgett et al., 2011), who identified ORPphilins as naturally occurring molecules targeting OSBP and ORP4L. These compounds, which include OSW-1, cephalostatin, stelletin, and schweinfurthin subfamilies, display cytotoxic and antiproliferative properties. Additional OSBP-inhibiting compounds have thereafter been identified and shown also to inhibit the replication of several, mainly positive-strand RNA viruses (Roberts et al., 2019; Strating and van Kuppeveld, 2017). Moreover, OSBP and other ORPs are hijacked by bacterial pathogens, including *Salmonella typhimurium* and *Legionella pneumophila* (Kolodziejek et al., 2019; Vormittag et al., 2023). Besides infectious diseases, OSBP/ORPs have been implicated in cancers (Olkkonen, 2021). The largest body of evidence has accumulated on ORP4L, the aberrant expression of which has been linked to leukemogenesis (Zhong et al., 2022; Zhong et al., 2019; Zhong et al., 2016).

Ongoing studies thus aim to develop specific inhibitors for the ORP family members for infectious diseases or cancers. Good examples are a schweinfurthin derivative targeting OSBP and having a distinct antitumor effect in a glioblastoma xenograft model when combined with the previously used drug temozolomide (Jezequel et al., 2023), and orpinolide, a synthetic analog that disrupts a leukemic dependency on cholesterol transport by inhibiting OSBP (Cigler et al., 2024). Likewise, an OSW-1 derivative with enhanced specificity for ORP4L eradicated acute myeloid leukemia stem cells in a xenograft model (Zhong et al., 2019). The development of specific ORP-targeting pharmaceuticals will undoubtedly open avenues to yet additional therapeutic opportunities.

## Concluding Remarks

**Figure 3.**
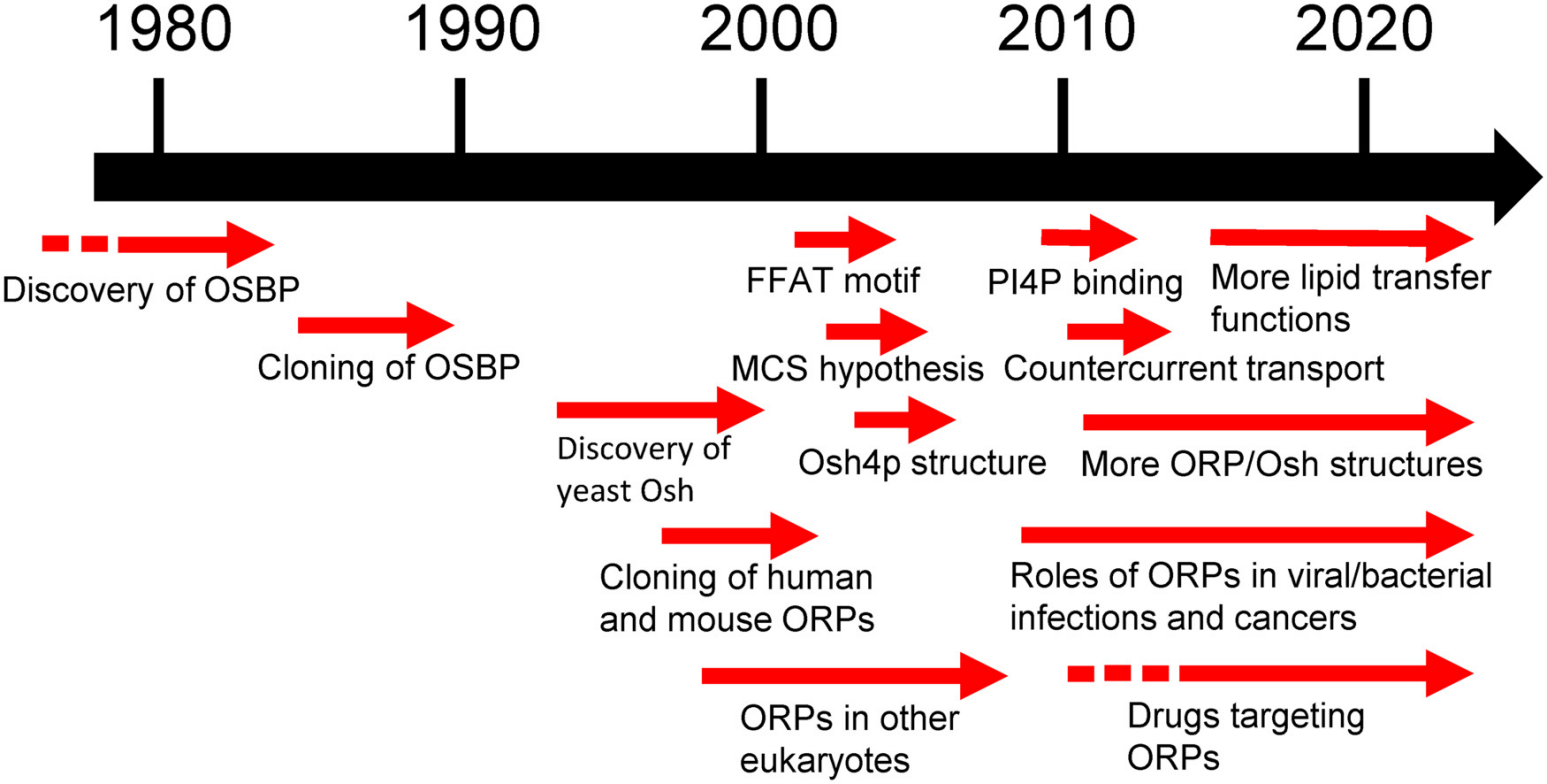
A timeline of key developments and discoveries in the study of OSBP/ORPs. Abbreviations: FFAT, two phenylalanines in an acidic tract motif; MCS, membrane contacts site(s); OSBP, oxysterol-binding protein; ORP, OSBP-related protein; PI4P, phosphatidylinositol 4-phosphate.

A timeline of key developments and discoveries in the study of OSBP/ORPs is depicted in Figure 3. OSBP was discovered approximately 40 years ago as an oxysterol receptor and assumed to act as a regulator of sterol homeostatic control. Families of its homologues (OSBP-related or -like proteins, ORPs/OSBPLs) were then identified in diverse taxa and considered as sterol receptors with functions that remained enigmatic for several years. With expanding structural and functional understanding these proteins have been recognized as central intracellular lipid transporters acting at specific MCSs. Their ligands are not limited to sterols but also contain glycerophosholipids: phosphoinositides, PI4P in particular, as well as PS.

However, the original idea of OSBP (or family members) executing intranuclear functions involving gene transcription can nevertheless hold, at least for the short OSBP-related proteins ORP1S and ORP2. Lee et al. (Lee et al., 2012) provided evidence that ORP1S regulates the LXR-dependent transcription of apolipoprotein E (APOE), while Escajadillo et al. (Escajadillo et al., 2016) reported a role of ORP2 in the transcriptional control of steroid hormone biosynthesis in adrenocortical cells. Thus, further investigation of the putative nuclear functions of ORPs seems warranted.

The current perception of ORPs as countercurrent transporters of lipids employing the energy of intracellular lipid gradients (Arora et al., 2022; Subra et al., 2023) was sparked by observations on ORP structure and lipid liganding (de Saint-Jean et al., 2011; Im et al., 2005) and by identification of VAP-related proteins as organizers of ER MCSs and recruiters of LTPs, including ORPs (Loewen et al., 2003). Structural studies on an increasing number of ORPs have provided insights into their lipid ligand specificities and transport mechanisms as well as interactions with membrane surfaces and protein interactions at MCSs. There is, however, so far little information on how the dynamic assembly and disassembly of higher order structures involving functional LTPs are coordinated. It is obvious that so far understudied regulatory regimes, including further post-translational modifications, are bound to play important roles.

A number of viral and bacterial pathogens hijack ORPs, and some of the family members are implicated in cancers. Therefore, ORPs are emerging as potential drug targets, and an important ongoing line of research is the development of specific inhibitors (perhaps also agonists) for distinct ORP family members. A next level of insight into ORP function necessitates (i) increasing knowledge on the structure of ORPs at their distinct conformational states and the lipid ligands and small-molecular antagonists that these proteins bind to, (ii) advanced genetically modified cell models for pinpointing ORP functions as parts of the endogenous cellular protein machineries and supramolecular complexes responsible for lipid transport and signaling, and (iii) tissue-specific and conditional knockout/inducibly expressing animal models, to analyze ORP functions in the physiologic context and in disease. Integrating data from all these levels of study will make it possible to place the ORPs into their full functional context in living organisms.
